# Mapping of Redox State of Mitochondrial Cytochromes in Live Cardiomyocytes Using Raman Microspectroscopy

**DOI:** 10.1371/journal.pone.0041990

**Published:** 2012-09-05

**Authors:** Nadezda A. Brazhe, Marek Treiman, Alexey R. Brazhe, Ninett L. Find, Georgy V. Maksimov, Olga V. Sosnovtseva

**Affiliations:** 1 Biological Faculty, Moscow State University, Russia; 2 Department of Biomedical Sciences, Copenhagen University, Copenhagen, Denmark; 3 The Danish National Foundation Research Center for Heart Arrhythmia, Copenhagen, Denmark; Université Joseph Fourier, France

## Abstract

This paper presents a nonivasive approach to study redox state of reduced cytochromes 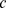, 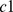 and 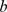 of complexes II and III in mitochondria of live cardiomyocytes by means of Raman microspectroscopy. For the first time with the proposed approach we perform studies of rod- and round-shaped cardiomyocytes, representing different morphological and functional states. Raman mapping and cluster analysis reveal that these cardiomyocytes differ in the amounts of reduced cytochromes 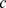, 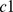 and 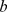. The rod-shaped cardiomyocytes possess uneven distribution of reduced cytochromes 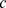, 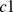 and 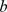 in cell center and periphery. Moreover, by means of Raman spectroscopy we demonstrated the decrease in the relative amounts of reduced cytochromes 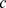, 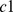 and 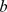 in the rod-shaped cardiomyocytes caused by H_2_O_2_-induced oxidative stress before any visible changes. Results of Raman mapping and time-dependent study of reduced cytochromes of complexes II and III and cytochrome 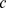 in cardiomyocytes are in a good agreement with our fluorescence indicator studies and other published data.

## Introduction

In respiring mitochondria, the continuous electron flow through the electron transport chain (ECT) down the redox potential span of approximately 1100 mV [1] allows the energy to be captured by a proton gradient, making a protonmotive force (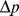) available for ATP production [2]. The redox state, or fractional reduction, of electron-conducting ETC components varies in response to a number of physiological or pathological factors, including ATP production rate, O_2_ availability, posttranslational modifications, mutations or damage of ETC proteins [3]. Conversely, high fractional reduction of ETC cytochromes may lead to structural and functional damage through augmented production of reactive oxygen species (ROS) (at complexes I and III), such as superoxide (O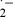) and H_2_O_2_, a source of hydroxyl radical (OH^·^) [3], [4]. For instance, during heart ischemia, excessive ROS generation appears to impair ETC function and prime mitochondria for further damage at reperfusion [5], [6]. ROS-induced oxidative stress is experienced by heart muscle in a number of additional conditions, including inflammation, heart failure and various cardiomyopathies [7] Hence, techniques to study redox state of ETC cytochromes *in situ*, and the relationships between their redox state, oxidative stress and their functional consequences in live, isolated cells or organs offer important insights into the role of mitochondria in a range of pathological conditions.

Live cell studies with Raman spectroscopy have been attracting an increasing interest because Raman spectroscopy provides information about the structure of biomolecules *in situ* in unlabeled cells and with no effect on the cell integrity [8]–[10]. Heme-containing cytochromes of the ETC possess an intensive Raman scattering depending on redox state of heme Fe [11]–[13]. Therefore, these cytochromes are excellent study objects for Raman spectroscopy. Raman studies of isolated cytochrome c and complexes II, III and IV demonstrated that depending on the excitation wavelength it is possible to obtain Raman scattering predominantly from cytochromes 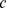 and 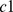 (cyt.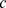, 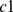), cytochromes 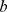 (cyt.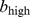, 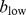 of complex III and cytochrome 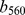 of complex II) or cytochromes 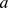 and 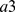. This Raman scattering strongly depends on the redox state of cyt.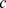 and complexes II, III, IV as well as on the potential of inner mitochondrial membrane (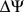) [11], [12], [14]. Distribution of mitochondrial cytochromes was visualized in HeLa [15] and yeast cells [16], using their characteristic Raman bands. Raman-based imaging can provide unique spatio-temporal information on mitochondrial function in intact cells. For instance, Raman microscopy was used to study the release of cytochrome 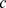 from mitochondria in HeLa cells under induced apoptosis [17]. Ogawa et al. [18] have shown that Raman spectra of cardiomyocytes (CM) from healthy and infarct regions of myocardium differ, and have attributed this to the different functional states of mitochondria. These authors have shown that peaks at 750 and 1125 cm^−1^ originate from cytochromes 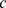, 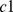 and 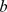 [18]. However, no further analysis of these peaks has been presented.

Here we propose a Raman-based approach for a semi-quantitative estimation of the amount of reduced cytochromes 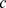, 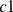 and 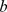 in isolated, live CM. We use this approach under two sets of experimental conditions: (i) employing well-characterized functional states of CM and (ii) in connection with H_2_O_2_ application to generate oxidative stress in CM. We also show that Raman images of CM are in a good agreement with corresponding images of CM stained with rhodamin 123 (Rh123), a fluorescent dye traditionally used to obtain information on the mitochondrial potential.

## Materials and Methods

### Cardiomyocyte preparation

The animal studies were conducted in accordance with international guidelines (National Institutes of Health publication no. 85-23, revised 1985 and Danish legislation governing animal experimentation, 1987), and were carried out after permission had been granted by the Animal Experiments Inspectorate, Ministry of Justice, Denmark. Single CM were prepared by enzymatic dissociation during retrograde perfusion of the heart using a modified Langendorff technique [19]. Briefly, rats were anesthetized with a mixture of hypnorm/midazolam/sterile water (1∶1∶2) by s.c. injection (0.25 ml/100 g body weight) and heparinized (5000 U/ml i.m., 0.7–1 ml/rat) for 10 min. Rat hearts were excised, mounted on a Langendorff system, and perfused through the aorta for 35 min using oxygenated, Ca^2+^-supplemented Tyrode solution containing (in mmol/L) 140 NaCl, 5.4 KCl, 10 HEPES, 1 MgCl_2_, 1 CaCl_2_, and 10 D-glucose (pH 7.4) at 37 deg C, then with Ca^2+^-free Tyrode solution for 7 min before digestion with 50 ml of the same solution containing collagenase (type 4, 50 mg, 300 units/mg) and protease (type XIV, 0.2 mg/ml), recirculated for 16–18 min. Collagenase was subsequently removed by perfusion with Ca^2+^-free Tyrode solution at pH 7.4 for 7 min. All solutions were gassed with 100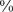 O_2_ for 5 min before use. The atria and blood vessels were then removed, and the free ventricles were minced into small pieces. Cells were dissociated by gentle mechanical shaking. Isolated CM were then incubated in Ca^2+^-free Tyrode solution with 1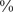 BSA and 20 mmol/L 2,3-butanedione monoxime (BDM) at room temperature for 20 min. The concentration of Ca^2+^ was gradually increased up to 1 mmol/L during the next 25 min. Cell viability and concentration were determined by Trypan blue assay. Typically, we obtained a 60–80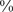 yield of quiescent, rod-shaped CM with the total yield from one heart 90–100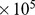 cells. Pyruvate 2 mM was added to the CM suspension before measurements, except in H_2_O_2_ studies. All chemicals were purchased from Sigma.

### Raman spectroscopy study

#### Raman imaging of cardiomyocytes

Raman imaging of cardiomyocytes was done by means of InVia Raman microscope (Renishaw, UK) with 532 nm laser in the streamline mode. 63× water immersion objective (Leica) with NA of 0.9 was used. Laser power on the sample stage was 3 mW. Raman spectra were registered in the region 1000–2000 cm^−1^ using 1800 lines/mm grating. The scanning region consisted of 20 (vertical)
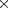
40 or 60 (horizontal) points, equivalent to 20
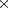
40 or 60 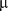
m. Raman spectra in points along a single line were recorded simultaneously. In each line the signal was collected for 15 s and the laser power in the scanning line was 3 mW. In experiments with H_2_O_2_, Raman spectra of CM were measured from a spot in CM center using the laser power 0.3 mW and 63× water immersion objective (Leica, NA 0.9). Signal collection was done for 20 s.

#### Analysis of Raman spectra and images

Images and spectra were processed using open source software [20]. First, baseline was subtracted at each image pixel. The parameters for baseline subtraction were chosen after processing of approximately 100 spectra from different cardiomyocytes to ensure that all baseline variations were taken into account. Raman images corresponded to an averaged intensity of the spectrum values within certain band interval, or to a ratio between specified peaks. For imaging we used 5 rod- and 4 round-shaped cells from two independent CM preparations. Results with H_2_O_2_ application were obtained from 10 cells from 9 independent CM preparations while control curves (without H_2_O_2_ application) were obtained through averaging over 5 cells from two independent CM preparations.

For cluster analysis of Raman images, we applied self-organizing map (SOM) algorithm [21], used widely in biomedical research [22], [23]. This algorithm maps data patterns onto an n-dimensional grid of units with similar behavior. Bacao et al [24] showed that the SOM algorithm outperforms a more widely used k-means clustering algorithm. Number of clusters (N = 6) was chosen as a minimal number resulting in a meaningful spatial structure. Simple Euclidean distance was used as a measure of distance between spectra. The algorithm started with random attribution of all spectra in the image to clusters, and then the spectra were iteratively re-attributed until no more than 1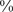 of spectra changed their attribution in a single iteration.

To plot Raman maps for cytochrome and oxy-myoglobin (oMb) peaks in CM, intensities of Raman peaks were determined for 750 cm^−1^, 1125 cm^−1^ and 1640 cm^−1^ bands for each pixel in the image. The resulting maps were used to visualize peak intensity ratios: 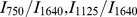 and 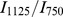, after areas outside the cells have been masked out. In H_2_O_2_ experiments, intensities of peaks at 750, 1125 and 1640 cm^−1^ at each time point were normalized to the intensity at time zero (before H_2_O_2_ or buffer application) or to the intensity of the peak at 1640 cm^−1^.

#### Fluorescent imaging of cardiomyocytes

To demonstrate difference in mitochondrial 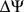 of different CM types we used rhodamin 123 (Rh123) in self-quenching mode (10^−6^ M) [25], [26]. CM were loaded with Rh123 in Tyrode solution for 10–12 min before microscopy. Rh123 fluorescence was excited with 488 nm laser and fluorescent spectra were registered in the region 530–567 nm. Fluorescent images were collected in a streamline mode of InVia Raman microspectrometer. 63× water immersion objective (Leica) with NA of 0.9 was used. Laser power on the sample stage was 0.15 mW. The scanning region consisted of 10 (vertical)×20 (horizontal) points corresponding to 10
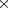
20 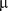
m. Fluorescence spectra were recorded simultaneously in points of each vertical line and the fluorescence signal accumulation was done for 2.5 s in each scan-line. To verify the self-quenching mode, FCCP (10^−5^–10^−6^ M) was used to dissipate mitochondrial potential and cause a fluorescence intensity increase by at least 50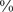 in Rh123-stained CM, owing to a decrease of Rh123 concentration in the mitochondrial matrix caused by the probe efflux.

## Results and Discussion

We performed experiments on CM in two well-described experimental systems. The first set of experiments was done on CM with different morphologies (rod-shaped and round CM), reflecting differences in cell intactness and energy state [27]. In the second set of experiments we studied redox state changes of mitochondrial cytochromes during early stage of oxidative stress caused by application of hydrogen peroxide (H_2_O_2_), when cell morphology was still not visibly affected.

### Peak assignment in Raman spectrum of cardiomyocytes

We first defined those bands of CM Raman spectra that were most sensitive to the redox state of cytochromes 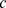, 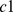 and 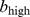, 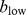 of complex III and cytochrome b of complex II (hereafter referred to collectively as cytochromes 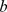). Green laser excitation causes resonance Raman scattering from heme-containing proteins: myoglobin (Mb), cytochromes 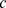, 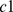 and cytochromes 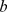, but not from cytochromes 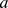, 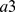 [12], [14]. Cytochromes 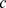, 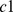, 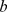 and Mb have a similar set of Raman peaks. However, since mitochondrial cytochromes are membrane-bound proteins and Mb is a free cytosolic protein, and since 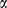 and 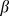 light absorption bands of cytochromes are slightly shifted with respect to those of Mb, positions and the relative intensity of their Raman peaks are also slightly different [11]. This makes it possible to separate cytochrome-associated peaks from Mb-associated peaks in the Raman spectrum of cardiomyocytes.

**Figure 1:**
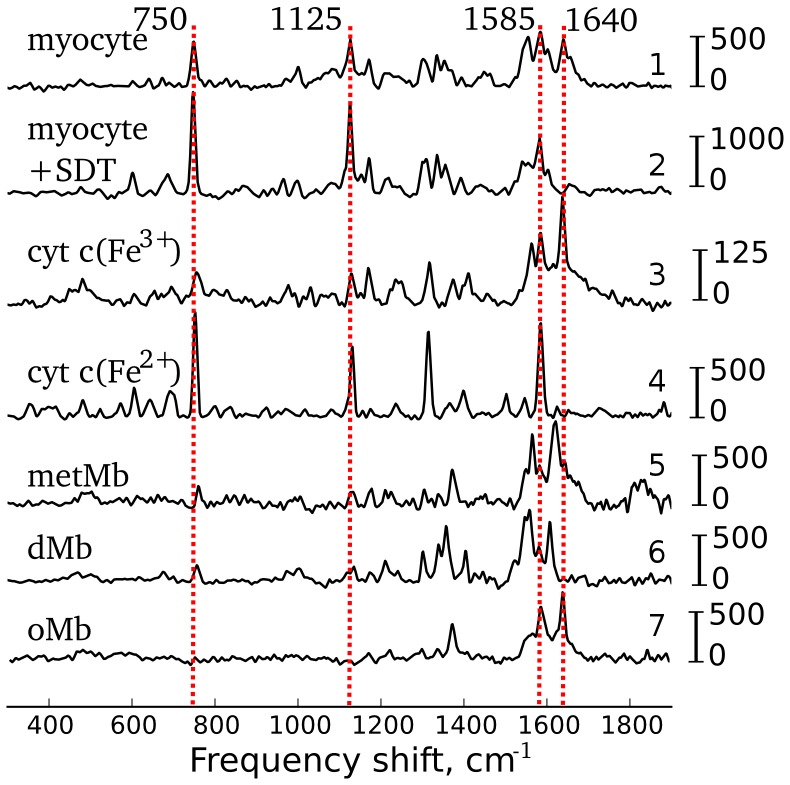
Raman spectral analysis. Raman spectra acquired from s freshly isolated cardiomycyte before (trace 1) and after (trace 2) application of sodium dithionite (SDT), oxidized (trace 3) and reduced (trace 4) cytochrome 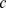, metmyoglobin (trace 5), deoxymyoglobin (trace 6) and oxymyoglobin (trace 7). Vertical scale bars near each spectrum show Raman intensity, a.u. Notice scale differences for trace pairs 1–2 and 3–4, visually attenuating the true differences between the oxidized and reduced states. All spectra are shown after baseline subtraction and are shifted vertically for clarity. Red dotted vertical lines show maximum positions of peaks of interest.

As the experiments were carried out under conditions without CM contraction, most of Mb molecules (no less than 90%) were expected to be in an oxygenated state [28]. At rest the respiration does not achieve its maximal rate and electron carriers are in partially reduced states. Figure 1 shows Raman spectra of a freshly isolated cardiomyocyte (traces 1 and 2), oxidized and reduced cytochrome 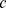 (traces 3 and 4, respectively), metmyoglobin (metMb), deoxymyoglobin (dMb) and oxymyoglobin (oMb) (traces 5, 6, and 7). Raman spectrum of cardiomyocytes had a multi-component structure, showing heme peaks of cytochromes 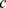, 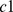, 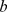 and Mb. We focused our study on the analysis of the most intensive peaks in cardiomyocyte spectrum: 750, 1125, 1585 and 1640 cm^−1^ — 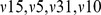 Raman bands, respectively.

**Peaks 750 and 1125 cm^−1^** are characteristic peaks in resonance Raman spectra of reduced cytochromes 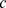 and 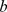 [11]–[13]. It is known that under the excitation in the region of absorption 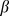-band (520–530 nm) Raman scattering of cytochromes 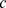 and 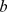 in oxidized form is very weak. Hence, in a mixture of reduced and oxidized cytochromes the intensity of the overall Raman scattering is defined by the reduced forms [12]. It has therefore been suggested that intensities of 750 and 1125 cm^−1^ peaks are related to the amount of reduced cytochromes 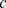 and 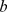 [13], [18]. One can see a large increase in the intensities of bands at 750 and 1125 cm^−1^ and of the whole Raman spectrum of cytochrome 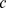 after reduction with sodium dithionite (SDT) (Fig. 1, traces 3 and 4). We observed a similar effect in cardiomyocytes. Application of SDT to cardiomyocytes caused reduction of previously oxidized cytochromes 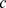 and 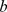 and hence intensities of peaks at 750 and 1125 cm^−1^ increased at least 3–4 times (Fig. 1, traces 1 and 2). Note that these peaks were only present as minor components in the Raman spectra of isolated dMb and metMb (but absent in spectra of oMb). Therefore, Mb contribution to the CM spectrum was negligible at these frequencies (Fig. 1). This provides evidence that peaks at 750 and 1125 cm^−1^ in CM spectrum originated from heme vibrations in cytochromes 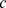 and 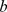. Adar et al. [11] demonstrated that with 530.9 nm laser excitation the Raman peak at 750 cm^−1^ was mostly determined by cytochromes 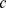, 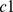, whereas the peak at 1125 cm^−1^ by cytochromes 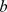.

**Peak at 1585 cm^−1^** was clearly expressed in Raman spectra of CM (with and without SDT application), in both forms of cytochrome c and in oMb (Fig. 1). Since Raman scattering of oxidized cytochromes is negligible, the peak at 1585 cm^−1^ originates from reduced cytochromes 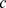, 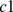 and 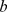 and oMb.

**Peak at 1640 cm^−1^** was present in the spectra of CM, oxidized cytochrome and oMb. It was absent in the spectra of reduced cytochrome, dMb and metMb (Fig. 1). Hence, peak at 1640 cm^−1^ in Raman spectrum of CM was due to oMb. Application of SDT caused deoxygenation of oMb and shifted 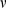
10 Raman band to 1603 cm^−1^ with vanishing peak at 1640 cm^−1^.

In summary, taking into account that Mb concentration in CM is at least 7 times higher than concentration of mitochondrial cytochromes [28] and based on the results in Fig. 1 and cited literature, we assign the discussed peaks as follows: (i) 750 cm^−1^ to reduced cytochromes 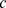, 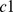 mainly; (ii) 1125 cm^−1^ to reduced cytochromes 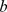 (cyt.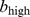, 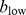 and 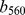) mainly; (iii) 1585 cm^−1^ to oMb and reduced cytochromes 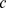; and (iv) 1640 cm^−1^ to oMb. Detailed assignment of other peaks in the Raman spectrum of individual cardiomyocytes is shown in Table 1.

**Table 1:** Positions and assignments of bands in Raman spectra of cardiomyocytes.*

Frequency, cm^−1^	Bond in heme molecule*	Symmetry of vibration*	Sensitivity of vibration*	Hemoprotein*	Main contribution from**
**747–750**	heme breathing	B1g, 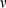 15	Redox state of heme Fe, 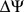	cytochromes 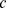 , 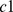 , 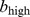 , 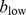 , 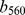 , oMb	cytochromes 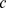 , 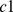
**1125**	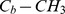	B1g, 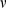 5	Redox state of cytochromes 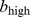 , 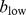 , 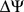	cytochromes 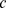 , 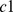 , 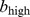 , 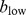 , 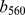 , oMb	cytochromes 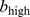 , 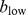 , 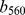
1170	asymmetric pyrrol half-ring	B2g, 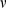 30	Redox state of heme Fe, 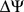	cytochromes 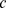 , 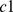 , 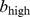 , 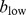 , 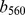 , oMb	cytochromes 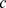 , 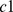 , 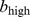 , 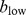 , 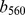
1297	all heme bonds	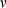 21	Redox and spin state of heme Fe, presence of O_2_	oMb	oMb
1301–1305	all heme bonds	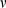 21	Redox state of heme Fe, 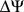	cytochromes 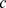 , 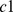 , 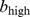 , 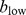 , 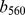	cytochromes 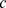 , 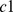 , 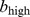 , 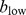 , 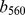
1335	all heme bond		Redox state of heme Fe, 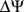	cytochromes 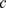 , 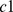 , 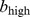 , 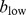 , 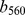	cytochromes 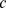 , 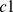 , 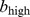 , 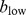 , 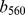
1370–1375	symmetric pyrrol half-ring	A1g, 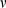 4	Redox state of heme Fe, presence of O_2_	oMb	oMb
**1585**	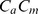 , 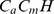	A2g	Redox and spin state of heme Fe, diameter of heme ring	cytochromes 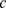 , 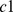 , 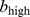 , 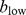 , 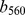 , oMb	cytochromes 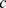 , 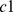 , 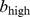 , 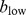 , 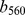 , oMb
**1640**	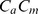 , 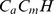 , 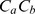	B1g, 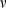 10	Spin state of heme Fe, diameter of heme ring	oMb	oMb

* Based on papers [11]–[13], [18], [35].

** Based on [12], [18] and our own observations. Frequencies indicated in bold type refer to the peaks discussed in the text.

In conditions when the amount and conformational state of oMb do not change the input of its Raman scattering to the overall CM Raman spectrum is constant. Therefore, under such conditions the oMb peak at 1640 cm^−1^ in CM Raman spectra can be used for normalization of intensities of the peaks at 750 and 1125 cm^−1^, to obtain a semi-quantitative estimation of the change in the amount of reduced cytochromes 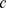, 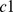 and 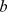.

### Morphological study of cardiomyocytes

Isolated, intact CM are elongated, rod-shaped cells with an appearance close to that of cells in a normal heart. In contrast, CM whose sarcolemma has been disrupted (as a side effect of the enzymatic digestion in the isolation procedure) may develop a characteristic, round shape [27]. In electron microscopy, these round CM have no regular sarcomeric structure. Instead, they present a hypercontracted, amorphous mass of myofibrils in the center, with mitochondria displaced towards cell periphery [27]. In a population of isolated CM, a spontaneous conversion of rod-shaped to round cells was observed with the proportion of the latter increasing from a few 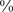 to about 50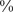 within one hour after isolation [27]. It is important to stress that these round CM, while severely dysfunctional, are not totally metabolically inactive. Indeed, a disinhibition of mitochondrial ATP production (in addition to glycolysis inhibition) was shown to induce a CM conversion into the round shape, from a distinct population of square, fully ATP-depleted cells [27]. Thus, while rod-shaped CM may be regarded as possessing normal rates of ATP synthesis, round CM are energetically compromised and likely to display lower rates of electron flux through ETC. We therefore studied Raman spectra of rod-shaped and round CM, with a view to detect redox state differences between their mitochondrial cytochromes.

**Figure 2:**
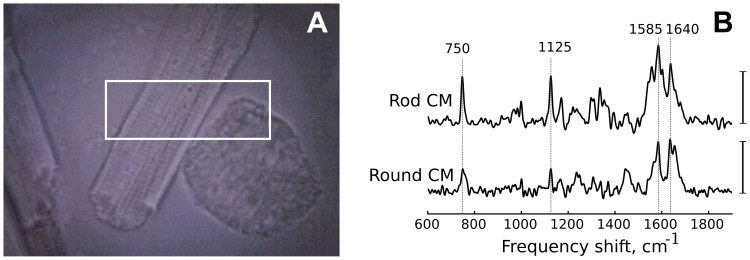
Rod- and round-shaped cardiomyocytes. (A) Microphotographs of rod- and round-shaped cardiomyocytes in the reflected light. White rectangle shows place on microphotographs where clustering and Raman maps shown in Figs. 3 and 4 were recorded. Horizontal length of the rectangle corresponds to 20 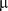
m. (B) Raman spectra of the same rod and round-shaped cardiomyocytes recorded from the cell centers. Vertical bar corresponds to the Raman intensity, a.u. Grey dotted vertical lines shows positions of main maxima in cardiomyocyte Raman spectra.

Figure 2 shows representative Raman spectra recorded from the center of each of the two cell types. Raman spectrum of the round cell is less intensive in the region 600–1400 cm^−1^, with weaker cytochromal peaks, compared to the spectrum recorded from the rod-shaped CM (Fig. 2 B). In addition, while oMb peaks at 1585 and 1640 cm^−1^ could be clearly distinguished in the round CM, the spectrum structure in this region (1550–1650 cm^−1^) differed markedly from that observed in rod-shaped CM.

**Figure 3:**
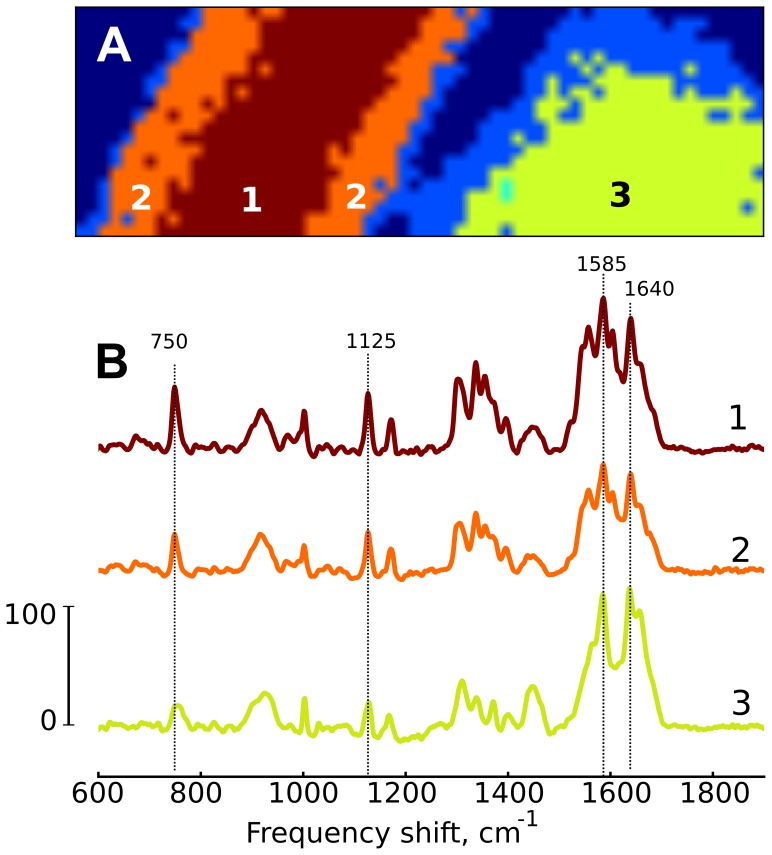
Raman cluster analysis. (A) Cluster maps of rod- and round-shaped cardiomyocytes from Fig. 2 A Each color corresponds to the individual cluster consisting of pixels that were defined to have similar Raman spectra. (B) Averaged Raman spectra for each cluster. Spectrum color and number correspond to the color and number of its cluster in panel A.

**Figure 4:**
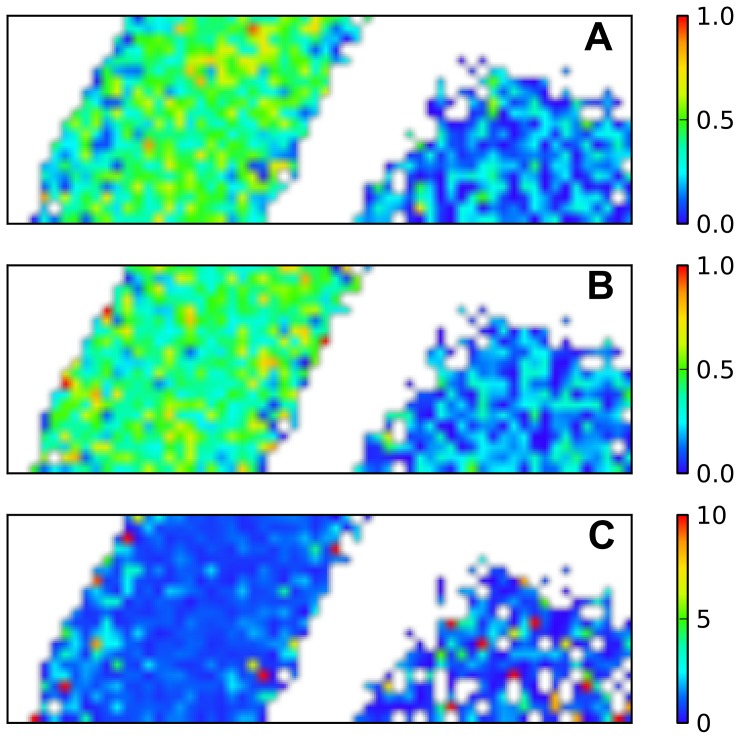
Raman map analysis. Raman maps of rod-and round-shaped cardiomyocytes from Fig. 2 A showing ratios 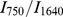 (A), 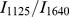 (B) and 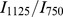 (C).

To further investigate the spectral properties of rod- vs. round-shaped CM we applied cluster analysis (method of self-organizing maps) [21]. This method generates clusters of similar spectra collected from a range of pixels (registration points). Figure 3 A shows the cluster map obtained for the same CM as in Figs. 2 and 4. Each color corresponds to a separate cluster, encompassing pixels with similar Raman spectra. This approach revealed that Raman spectra in the rod-shaped CM could be assigned to two different clusters represented in Fig. 3 (brown (1) and orange (2) colors, respectively), while spectra in the round CM could be assigned to a third, distinct cluster (yellow color (3)). Pixels in boundary regions of both CM types were assigned to the fourth cluster category, likely reflecting the low Raman scattering intensity in boundary areas. Figure 3 B shows averaged Raman spectra for these clusters, using colors and numbers corresponding to cluster images in Fig. 3 A. It may be seen that averaged Raman spectrum from round CM has a relatively low representation of “cytochromal” peaks at 750 and 1125 cm^−1^ (trace 3, yellow). Besides, the spectrum structure in the region at 1200–1400 cm^−1^ for the averaged Raman spectra of round CM differs from the same region in Raman spectra of both center and periphery in the rod CM. Moreover, the group of peaks in the region 1550–1670 cm^−1^ of the round CM has different shape compared to peaks in the rod-shaped CM.

In order to quantify the spectral differences between the two regions of the rod CM and between both regions of rod CM and round CM we determined within each cluster mean values and 95% confidence intervals for peak intensity ratios: 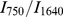, 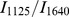 and 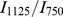. Such “internal normalization” of certain Raman peak intensities on the intensity of the chosen reference peak insures that comparison of different cells or different regions in the same cell can be done correctly without effect of possible focus shift or of the different concentration of studied molecules in various cells/cell compartments that influence absolute values of peak intensities.

If it is assumed that the conformation and relative concentration of oMb in the rod-shaped CM is the same throughout the cell then ratios 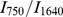 and 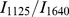 would give a semi-quantitative estimation of the amount of reduced cytochromes 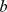 and 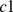, 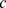. The performed analysis showed a statistically significant enrichment in the amount of reduced cytochromes 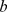, 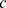 and 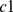 relative to oMb in the center region (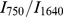 and 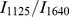, Table 2). The ratio 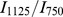 was found to be significantly lower in the cell center (Table 2). This finding could indicate the higher amount of reduced cytochromes 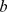 relative to the reduced cytochromes 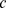 in the cell periphery. Only one spectral region was distinguished by the cluster analysis of round CM (Fig. 3 A). Peak intensity ratios relative to oMb for the round cell are shown in Table 2. These ratios showed an approximately 50% decrease, when compared to the same peak ratios in any region of the rod-shaped cell. There is no literature data about Mb concentration in the round-shaped CMs, however, it seems quite possible that oMb amount is approximately the same as in rod-shaped CMs. Therefore, the observed spectral difference between regions of the rod and round CMs could be attributed to the decreased amount of reduced cytochromes 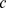, 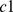 and 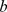 in complexes II and III of mitochondria in the round-shaped CM.

**Table 2:** Mean values and 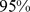-confidential range (shown in brackets) for ratios 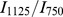, 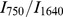 and 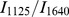 calculated for clusters in rod-and round-shaped cardiomyocytes shown in Fig. 3.

Ratio	Center of rod CM (brown cluster)	Periphery of rod CM (orange cluster)	Round CM (yellow cluster)
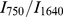	0.47 (0.46, 0.48)*^a^*	0.38 (0.36, 0.39)*^a^*	0.19 (0.18, 0.19)*^a^*
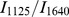	0.42 (0.41, 0.43)*^b^*	0.4 (0.38, 0.41)*^c^*	0.19 (0.18, 0.20)*^b,c^*
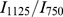	0.89 (0.86, 0.93)*^d^*	1.05 (0.99, 1.12)*^d^*	1.02 (0.95, 1.09)

Nonparametric Kruskal-Wallis test with post Dunn's multiple comparison test. Upper indexes  indicate groups between which there is a significant statistical difference with 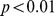.

The results from Table 2 may be visualized using color-coded maps of peak intensity ratios, shown in Fig. 4. To produce such maps pixel-by-pixel ratio images were obtained using appropriate frequency pairs. In the rod-shaped CM, the central enrichment (relative to oMb) of reduced cytochromes 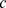 and 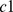 (corresponding to 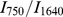; Fig. 4 A) and of reduced cytochromes 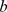 (corresponding to 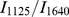; Fig. 4 B) can be seen contrary to a slightly higher ratio of the amount of reduced cytochromes 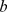/reduced cytochromes 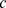 at the cell periphery (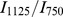; Fig. 4 C). In the round cell, the low amounts of reduced cytochromes 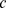, 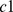 and 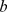 compared to the rod-shaped cell are apparent (Figs. 4 A and B).

The distribution of reduced cytochromes 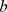, 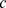 and 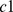 within the rod-shaped CM in a manner identifying a central and a peripheral region correlates with the known heterogeneity of mitochondria in these cells. Indeed, the centrally located, interfibrillar mitochondria are thought to be enriched in cytochromes 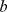 and 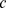, as compared to the peripherally located, subsarcolemmal mitochondria [29]. However, our data does not distinguish between the amounts of reduced cytochromes per unit mitochondrial protein vs amounts in total mitochondrial volume scanned. Studies on preparations of separated interfibrillar and subsarcolemmal mitochondria will be necessary to determine, whether these differences in the amounts of reduced cytochromes are inherent to the two mitochondria categories.

In the round CM, a separation into regions with differing spectral properties was absent (Fig. 3 A, Fig. 4 A–C). This finding is consistent with the electron microscopy data, showing an amorphous sarcomere mass (contraction band) surrounded by mitochondria scattered in a manner precluding any spatial separation of the interfibrillar and subsarcolemmal mitochondria [27]. Round CM are energetically compromised cells, capable of some residual oxidative ATP synthesis [27]. To our knowledge, the present results represent the first demonstration of a markedly diminished amounts of reduced cytochromes 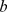, 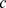 and 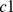 in these cells when compared to normal, rod-shaped CM (Table 2, Fig. 3 A, Fig. 4 A and B).

**Figure 5:**
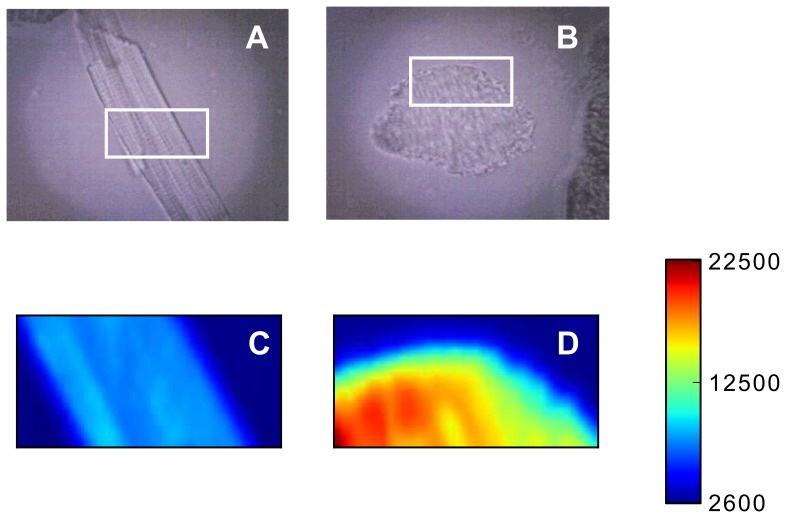
Fluorescent images. Microphotographs of (A) rod- and (B) round-shaped cardiomyocytes in the reflected light and fluorescent images of the same cells obtained with 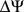-sensitive dye rhodamin123 (C) and (D). Increase in the intensity of Rh123 fluorescence corresponds to the decrease in 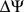. Color bar shows value of the fluorescence intensity in cps. White rectangles on microphotographs show cell areas where Raman images were recorded. Horizontal length of the rectangles corresponds to 20 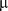
m.

It is known that redox state of mitochondrial cytochromes is influenced by transmembrane potential [30], [31]. In order to test the association between the low amounts of reduced cytochromes 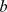, 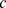 and 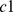 in round CM and their mitochondrial membrane potential 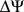 we performed fluorescent imaging of rod-shaped and round CM using Rh123. Rh123 fluorescence intensity in the rod-shaped CM was clearly smaller than in the round cell (Fig. 5 C and D). Since we used Rh123 in the self-quenching mode (see Materials and Methods), this difference was due to a higher accumulation of the probe in the mitochondria of the rod-shaped cell, reflecting their higher 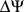 value as compared to 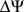 in the round cell mitochondria. We conclude that the present data on the decreased amounts of reduced cytochromes 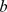, 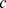 and 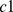 and low mitochondrial membrane potential in round CM are consistent with the low ATP synthesis activity found in these cells [27].

### Raman study of cardiomyocytes under oxidative stress

Treatment with H_2_O_2_ is widely used to simulate oxidative stress in CM under various pathological conditions. For instance, upon start of reperfusion following heart ischemia, a rapid O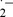 production at mitochondrial complexes I and III results in some of this O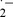 being converted to H_2_O_2_ [3], [32]. It is known that application of H_2_O_2_ causes a decrease in the activity of respiratory complexes, a decrease in 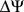 and ATP synthesis, eventually followed by mPTP opening [33]. H_2_O_2_ induces ROS formation by Fenton reaction, in which Fe^2+^ ion is oxidized by H_2_O_2_ with generation of the aggressive hydroxyl radical (OH). 2Fe/2S center of complex III is among sites of OH^·^ production [1]. Mitochondrial cytochromes may, therefore, be anticipated to be vulnerable to oxidative damage caused by OH^·^ generation in their near proximity. In order to test whether such damage would be detectable in their Raman spectra, we performed investigation of H_2_O_2_ effect on rod-shaped CM.

**Figure 6:**
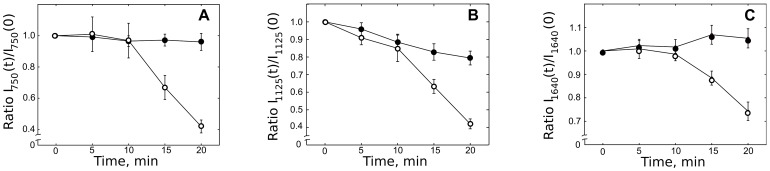
Effect of H_2_O_2_ on freshly isolated rod-shaped cardiomycytes. Effect of H_2_O_2_ on the intensities of Raman peaks at 750 (A), 1125 (B) and 1640 cm^−1^ (C) of freshly isolated rod-shaped cardiomycytes (open markers). Zero point of time corresponds to the values of spectra recorded before H_2_O_2_ application. Closed markers show time-dependence of the same peak intensities in control experiments with application of Tyrode buffer instead of H_2_O_2_. Values are mean 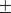 SEM. Nonparametric Kruskal-Wallis test with post Dunn's multiple comparison test gives 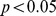 between results for 0 min and 15–20 min.

**Figure 7:**
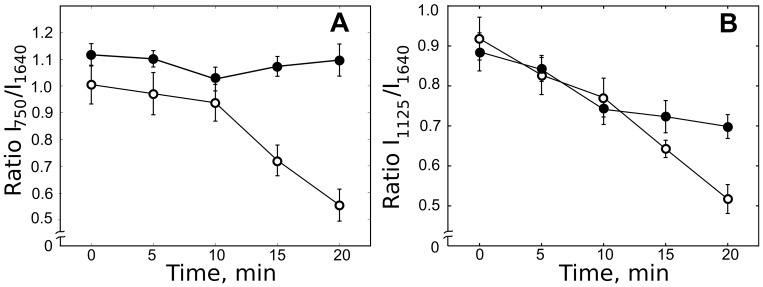
Effect of H_2_O_2_ on the amonut of reduced cytochromes. Effect of H_2_O_2_ on ratios 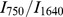 and 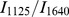 semi-quantitatively corresponding to amounts of reduced cytochromes 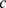, 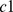 (A) and cytochromes 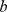 of complexes II and III (B), respectively. Zero point of time corresponds to the ratios of spectra recorded before H_2_O_2_ application (open markers). Closed markers show time-dependence of the same peak ratios in control experiments with application of Tyrode buffer instead of H_2_O_2_. Values are mean 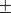 SEM. Nonparametric Kruskal-Wallis test with with post Dunn's multiple comparison test gives 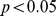 between results for 0 min and 15–20 min.

H_2_O_2_-treatment of CMs did not appear to cause any changes in the number or position of Raman peaks. However, a decrease of peak intensities at 750, 1125 and 1640 cm^−1^ in the H_2_O_2_-treated cells was seen within the 20 min of observation time (Fig. 6). Notably, intensities of “cytochromal” peaks at 750 and 1125 cm^−1^ decreased by approximately 60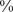, or twice as much as the intensity of oMb peak at 1640 cm^−1^. This faster decline of the 750 and 1125 cm^−1^ peak intensities relative to that of the 1640 cm^−1^ peak may also be seen in Fig. 7, in which the 1640 cm^−1^ peak values at each time point were used for normalization. The observed decrease in the intensities of peaks at 750, 1125 and 1640 cm^−1^ likely reflects combined effects of heme oxidation and irreversible oxidative damage to cytochrome and myoglobin heme-protein structures. H_2_O_2_ is known to react with myoglobin in ferrous and ferric states to generate ferryl myoglobin, in turn giving rise to Mb-X, a cross-linked form of heme-myoglobin [34]. Ferryl myoglobin is also known to propagate the oxidative damage by reacting with lipids to form lipid radicals [34]. The observed decline in the intensities of peaks at 750, 1125 and 1640 cm^−1^ was not evoked by laser-induced photodamage. Thus, in control experiments where CMs were exposed to laser irradiation with the same power but not to H_2_O_2_, peak intensities remained constant (Fig. 6 A and C) or showed small decrease (Fig. 6 B).

Figures 6 and 7 reveal an approximately 10 min lag before a H_2_O_2_-induced strong decrease in peak intensities ensured. However, even after 20 min of incubation with H_2_O_2_, when the decline in the Raman peak intensities was pronounced, the cells continued to display a rod-shaped form and a non-disturbed sarcomere structure, with no alteration in the morphology at the light microscopy level. Akao et al have shown that H_2_O_2_ treatment of cultured, neonatal CM lead to a progression of mitochondrial swelling (priming stage with duration of 20–40 min), depolarization due to mitochondrial permeability transition (with duration of 4 min) and fragmentation [33]. While a precise relationship of these stages to our observations cannot be ascertained at present, it would appear that Raman spectroscopy (Figs. 6 and 7) provided a relatively early information on the effects of H_2_O_2_ treatment.

## Conclusion

We report here an application of *in vitro* Raman microspectroscopy to study redox state of mitochondrial cytochromes of complexes II and III and cytochrome 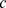 in live isolated cardiomyocytes. Since heme-containing cytochromes of mitochondrial respiratory chain give rise to an intensive Raman scattering, Raman technology gives a unique opportunity to study mitochondrial function in intact live cells. Excited by green laser light, reduced cytochromes give Raman scattering with the intensity of about 10 times higher than their oxidized forms. Therefore, Raman signal from a mixture of cytochromes in different redox states may be considered to originate predominantly from reduced cytochromes. In CM, myoglobin is another heme-containing protein contributing to the Raman scattering. We propose that under conditions when conformation and relative amount of oMb is constant, Raman peak at 1640 cm^−1^ may be used as an internal reference to evaluate a relative contribution of cytochromal peaks at 750 and 1125 cm^−1^ to the CM Raman spectra. Such internal normalization makes it possible to estimate, in a semi-quantitative fashion, changes in the amount of the reduced cytochromes 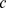, 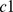 and 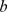.

We performed two series of experiments in well-described systems: (i) rod-shaped and round CM, differing in their energy status [27], and (ii) rod-shaped CMs treated with H_2_O_2_, known to affect mitochondria as such and complex III in particular [3], [32]. Raman mapping and clustering analysis in accordance with the proposed peak ratios in rod-shaped and round CM revealed significant differences between cell types, attributable to the different state of mitochondrial cytochromes. Rod-shaped CM possessed intensive peaks at 750 and 1125 cm^−1^, when compared to oMb peak at 1640 cm^−1^ (Figs. 3 and 4). This spectral profile implied a high amount of reduced cytochromes 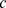 and 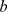, consistent with the normal function of the respiratory chain and ATP synthesis in rod-shaped CM. In contrast, energetically compromised, round-shaped CM lacked Raman scattering of reduced cytochromes in most areas, or Raman scattering intensity was low because of oxidation of all electron carriers of the respiratory chain. The results of Raman experiments on rod-shaped and round CM were in agreement with the data of fluorescent microspectroscopy with 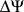-sensitive dye Rh123 (Fig. 5). Using clustering analysis we also demonstrated that distribution of reduced cytochromes 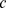, 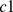 and 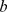 in rod-shaped CMs is uneven with higher relative amount of reduced cytochromes 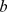 and 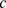 in the cell center (Fig. 3). Further, we showed that application of H_2_O_2_ caused a significant decrease in the amount of reduced cytochromes 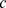 and 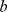 within 15–20 min with a 10 min time lag, consistent with published biochemical studies [33].

To conclude, we demonstrated that Raman microspectroscopy utilizing 532 nm laser light is a promising technique to obtain semi-quantitative information about amount of reduced cytochromes 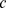, 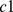, and 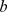 of complexes II and III in live, isolated CMs. The advantage of the proposed Raman-based approach is that it does not require any CM modification or staining with fluorescent dyes, and is performed *in situ*, without isolation of respiratory complex.
